# Pain and Communication in Children with Cerebral Palsy: Influence on Parents’ Perception of Family Impact and Healthcare Satisfaction

**DOI:** 10.3390/children8020087

**Published:** 2021-01-27

**Authors:** Inmaculada Riquelme, Álvaro Sabater-Gárriz, Pedro Montoya

**Affiliations:** 1 Research Institute of Health Sciences (IUNICS-IdISBa), University of the Balearic Islands, 07122 Palma, Spain; alvaro.sabater@uib.es (Á.S.-G.); pedro.montoya@uib.es (P.M.); 2 Department of Nursing and Physiotherapy, University of the Balearic Islands, 07122 Palma, Spain; 3 Fundación Aspace Baleares, Ctra. Vieja de Bunyola, Km 8.2, 07141 Marratxí, Spain

**Keywords:** cerebral palsy, pain, speech, family impact, healthcare satisfaction

## Abstract

Cerebral palsy (CP) is an impacting chronic condition. Concomitant comorbidities such as pain and speech inability may further affect parents’ perception of the pathology impact in the family quality of life and the provided care. The objective of this cross-sectional descriptive correlational study was to compare parental reports on family impact and healthcare satisfaction in children with CP with and without chronic pain and with and without speech ability. Parents of 59 children with CP (age range = 4–18 years) completed several questions about pain and speech ability and two modules of the Pediatric Quality of Life Measurement Model: The PedsQLTM 2.0 Family Impact Module and the PedsQLTM Healthcare Satisfaction Generic Module. Our findings revealed that children’s pain slightly impacted family physical health, social health and worry. In children without pain, speech inability increased the perceived health impact. Parents’ healthcare satisfaction was barely affected by pain or speech inability, both increasing parents’ satisfaction in the *professional technical skills* and *inclusion of family* domains on the care plan. In conclusion, pain and speech inability in children with CP can impact family health but not healthcare satisfaction. Regular assessment and intervention in family health is essential for the design of family-centred programs for children with CP.

## 1. Introduction

Children with cerebral palsy (CP) have unique demands, causing significant impact to the quality of life of their families. The care that people with cerebral palsy require throughout their lives involves a great financial burden, a significant investment of time and significant repercussions on work and social activities [1,2,3]. Undoubtedly, all this can generate chronic stress in the family and caregivers who take care of these persons [4,5], thus compromising their health and well-being [3,6,7]. Thus, parents of children with CP have reported poorer physical and mental health than the general population, with higher levels of depression, musculoskeletal pain and fatigue [7,8,9,10,11,12]. Furthermore, the family impact does not appear to be associated with the dependency level, age, type or severity of CP [4,10,11,13]. Even improvements in the child’s motor function do not produce changes in the quality of life of the parents [14]. Rather, parents develop negative feelings due to reductions or difficulties in the health, social skills, behaviours or emotions experienced by their children [4,15,16]. In this context, pain may be an important factor influencing family well-being, as concern for the child’s pain is one of the most reported causes of emotional stress in parents of children with CP [13,17].

More than half of children with CP experience frequent moderate to severe pain at multiple body locations [18,19]. Recurrent pain produces an increase of behavioural and emotional problems in children with CP, reducing their quality of life and negatively affecting their participation in daily and social activities and the satisfaction of parents with performing these activities [20,21,22]. Pain constitutes an additional burden for the health system, producing more demand for health services than the severity of the pathology [23], with more frequent visits to the family physician, more prescription of analgesics [18] and more recurrent use of conventional and alternative therapies [24]. Furthermore, pain is one of the main concerns of parents when their children are faced with a health intervention [25] and one of the main factors influencing parents’ evaluation of the intervention success [26]. Therefore, frequent pain reduces satisfaction with motor rehabilitation, while a low level of post-operative pain increases satisfaction after recovery and effective pain management is considered to improve the quality of healthcare [27,28].

The child’s inability to speak is an important risk factor for parental stress and depression [17,29], increasing the vulnerability perceived by parents in interventions that affect health, such as surgery [30], and poor perception of the child’s health-related quality of life [31]. Although parents are able to detect pain in their children in spite of their speech impairments [32,33], parent report increased frequency, duration and intensity of musculoskeletal pain in more severely affected children who are unable to self-report [34]. Moreover, discrepancies between parents and health professionals in pain detection are greater in children with speech problems [32].

Although pain and speech are important factors affecting parents’ quality of life and satisfaction with health services, little research has focused on their specific associations. This study aims to compare parental reports on family impact and healthcare satisfaction in children with cerebral palsy with and without chronic pain, as well as with and without speech ability.

## 2. Materials and Methods

### 2.1. Participants

This is a cross-sectional descriptive correlational study, with purposive sampling and a survey method for data collection. The staff, which is responsible for 11 specialized centres dedicated to education, care or leisure for individuals with disabilities in Majorca (Spain), identified the participants with cerebral palsy. The inclusion criterion was a diagnosis of CP and age between 4 and 18 years. Most of the participants were identified in care centres for children with cerebral palsy (80% of families), while a smaller percentage were identified in educational or leisure centres that support different developmental conditions. The parents of 70 children with CP were initially contacted through a letter explaining the objectives and protocol of the study. Moreover, informative meetings were held with the families at the centres to explain the objectives and methods of the study throughout the last quarter of 2016. Parents of 59 children with CP (age range = 4–18, mean age = 11.58 (4.61), 34 girls) agreed to participate in the study and provided written informed consent. In addition, children with CP with a sufficient cognitive level expressed verbal or gestural willingness to participate. The study was approved by the Ethics Committee of the Regional Government of the Balearic Islands (Reference code: IB3156/16 PI).

### 2.2. Interview and Questionnaires

The week following the signing of the informed consent, each family was assigned an anonymous code for data collection. Parents completed a semi-structured interview, and two 2-report questionnaires were delivered to be completed at home. The semi-structured interview (Supplementary Table S1) consisted of several questions about demographic data (Table 1), as well as about the pain and communication characteristics of their children. A member of the research team was in permanent telephone contact with the families to resolve possible doubts while the questionnaires were being completed. Parents were asked to return the completed questionnaires to the centre in a sealed envelope, and a member of the research team collected them each week. The type of CP, the cognitive level and the level of motor impairment, determined by the Gross Motor Function Classification System (GMFCS-R) [35], were obtained from the children’s medical history. Table 2 displays the clinical characteristics of children with CP.

Children’s pain was measured using the following information from the interview: (1) Whether they were experiencing chronic pain (pain lasting more than 3 months) or not (yes/no response); (2) ratings of current and worst pain in the last week using a 11-point numerical rating scale (0 = no pain, 10 = unbearable pain); and (3) location of painful body regions using a human figure drawing (QL07/00 Pediatric Pain Questionnaire) [36]. Speech ability was assessed with a yes/no question.

Parents completed two questionnaires included in the Pediatric Quality of Life Measurement Model (PedsQL^TM^, Lyon, France) [37] to assess family impact and healthcare satisfaction. The PedsQL^TM^ 2.0 Family Impact Module consists of 36 items comprising 8 dimensions: *Physical functioning*, *emotional functioning*, *social functioning*, *cognitive functioning*, *communication*, *worry*, *daily activities* and *family relationships*. Items are rated on a Likert scale from 0 (never) to 4 (almost always) and are transformed into a score from 0 to 100, with higher scores indicating better functioning (less impact). The 8 dimensions are combined into 3 total scores: The *Total impact score*, *Parent health-related quality of life summary score* and *Family functioning summary score*. The PedsQL^TM^ Healthcare Satisfaction Generic Module consists of 24 items comprising 6 dimensions: *Information*, *inclusion of family*, *communication*, *technical skills*, *emotional needs* and *overall satisfaction*. Items are rated on a Likert scale from 0 (never) to 4 (always) and are transformed into a score from 0 to 100, with higher scores indicating greater satisfaction. The PedsQL^TM^ model and its different questionnaires have proven to be valid and reliable for assessing different aspects of paediatric health-related quality of life [36].

### 2.3. Statistical Analyses

Multivariate analyses of variance (MANOVA) were performed on family impact and healthcare satisfaction separately. The factors PAIN (children with pain vs. children without pain) and SPEECH (children with speech ability vs. children without speech ability) were used as between-subject factors in the statistical design. In addition, the factor DIMENSION was used as within-subjects to assess effects on the different subscales of each module. In case of significant effects due to DIMENSION, separate ANOVAs on the scores of each subscale were planned to further explore the differences due to PAIN and SPEECH. All results were adjusted using Bonferroni corrections for post-hoc comparisons. In addition, Pearson and Spearman correlations were performed to establish associations among the different dimensions of the family impact and healthcare satisfaction questionnaires with pain characteristics. The missing data were not replaced or completed by statistical methods and were discarded from the analyses. Significance levels were set at *p* < 0.05.

## 3. Results

Once the recruitment performed, the sample size was estimated at 30 children. Pain was reported in 51% of the children (*n* = 30), and 40.7% had speech problems (*n* = 24). Parents reported moderate impact (mean = 69.14 (17.03), range = 26.39–96.53) and healthcare satisfaction (mean = 64.96 (21.94), range = 2–100). The descriptive data of the different dimensions for each of the four groups are displayed in Table 3.

**Figure 1:**
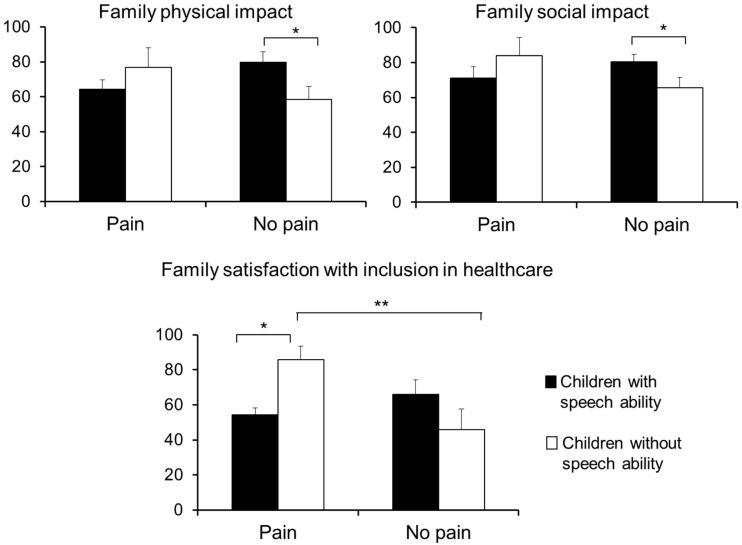
Mean and standard errors of the four groups of children in the significant dimensions of the Family Impact and Healthcare Satisfaction modules. * *p* < 0.05, ** *p* < 0.01.

In the Family Impact Module questionnaire, the MANOVA revealed only a main effect due to DIMENSION (F (7,27) = 17.77, *p* < 0.001), indicating that scores in the different subscales were significantly different. To further explore this effect, separate ANOVAs were performed on each dimension to examine the effects due to PAIN and SPEECH (Table 4). For the *physical functioning* dimension, a significant effect due to PAIN × SPEECH (F (1,39) = 5.04, *p* = 0.031) was yielded, indicating that parents of children without speech ability reported lower scores (higher negative impact) than those of children with verbal speech when children have no chronic pain (*p* = 0.044) (Figure 1). By contrast, no differences due to speech ability were observed on physical impact when children have chronic pain (*p* = 0.268). For the *social functioning* dimension, a significant interaction PAIN × SPEECH (F (1,39) = 4.38, *p* = 0.044) was also found, revealing that parents of children without speech ability reported lower scores (higher impact) than those of children with verbal speech when children report no chronic pain (*p* = 0.087) (Figure 1). No differences due to speech ability were observed on social functioning when children report chronic pain (*p* = 0.260). No other significant effects were found in the rest of the domains.

In addition, the number of pain locations correlated negatively with scores in the *worry* dimension of the family impact (r = −0.37, *p* = 0.033), indicating increased parents’ worry (lower functioning) when the number of painful body locations increased. The worst pain during the week correlated with the *communication* dimension (r = 0.515, *p* = 0.029), revealing lower impact in family communication with higher intensity of the child’s pain.

In the Healthcare Satisfaction Module questionnaire, the MANOVA revealed a main effect due to DIMENSION (F (5,27) = 6.17, *p* < 0.001), indicating that scores in the different subscales were significantly different. In order to further explore differences due to PAIN and SPEECH, separate ANOVAs were performed on the scores of these subscales (Table 4). In the *technical skills* dimension, a significant main effect due to SPEECH (F (1,36) = 6.80, *p* = 0.014) revealed higher satisfaction in parents of children without speech ability compared with parents of children with verbal speech. In the *inclusion of family* dimension, a significant interaction PAIN × SPEECH (F (1,37) = 8.24, *p* = 0.007) indicated that (1) parents of children without speech ability reported higher satisfaction when children report chronic pain than when they report no pain (*p* = 0.009), whereas no differences were observed in children with verbal speech ability (*p* = 0.293); and (2) parents of children with pain reported higher satisfaction when children did not have speech ability than when they have speech ability (*p* = 0.021), whereas no differences were found in children without chronic pain (*p* = 0.113) (Figure 1). There were no other significant ANOVA effects or significant correlations regarding parents’ healthcare satisfaction.

## 4. Discussion

The objective of the present study was to explore the mutual influence of children’s pain and speech ability on parental perception about the family impact and healthcare satisfaction in children with cerebral palsy. Our findings point to a slight impact of pain on family functioning. Only a few dimensions, such as *physical functioning*, *social functioning* and *worry*, seem to be affected by the presence of pain, which modulates the least perceived impact when the child has verbal speech. Parental satisfaction with healthcare was barely affected by pain or the lack of speech, increasing both the parental satisfaction with professional technical skills and inclusion in the plan of care.

Pain affected physical and social family functioning and the number of pain locations impacted on parental worrying. Pain has been reported to be a factor that increases the demand for care also in other chronic paediatric pathologies such as osteogenesis imperfecta [38]. Other studies have reported a good parental understanding of children’s expressions of pain, even when they cannot communicate verbally [32,39]. In the present study, we observed that families of children with chronic pain (with and without speech abilities) reported equal impact on physical and social health. A periodic evaluation of the physical, social and psychological status of the parents should be included in the protocols of the family-centred care models for children with CP in order to detect the specific areas (e.g., worry about child’s pain) that deserve specific attention [10,16,23]. In this sense, some experiences, such as web-based intervention programs that provide training in daily care to mothers of children with CP or home-based programs that use augmentative and alternative communication, have proven to improve the experience of care and quality of life of the caregiver [40,41]. Interestingly, the greater intensity of the children’s pain produced a lesser impact on family communication. Other studies with challenging situations, such as chronic life-threatening illnesses, have shown that parents concentrate on solution-focused communication, deferring potentially distressing discussions [42]. Thus, it seems that the presence of pain can help to promote pragmatic communication among family members to solve critical problems.

Inclusion in healthcare decisions, encompassing all phases of assessment, intervention and evaluation, is a critical determinant of high-quality care for parents and chronically ill children [43,44]. Families understand inclusion as the ability to communicate, understand the care plan and participate with the health team in decision-making [45]. Another factor that promotes family satisfaction with healthcare is professional competence [46], which is defined in a complex way and encompasses the attributes of emotional and communication skills (providing empathy for child/family, explaining procedures, answering questions) [47,48,49]. By contrast, misunderstanding of the problem or differences in intervention priorities negatively affect the parent-professional relationship [25,50]. Challenging situations, such as a lack of verbal speech or pain, require focusing on the problem, and require health professionals to improve their competences, reinforcing parental satisfaction with healthcare.

Limitations. The questionnaires were answered by one of the parents (mostly the mother), even in divorced families. Therefore, the perception of the other partner may differ. Our sample was small and biased toward participants with high motor difficulties (76.3% of the sample had GMFCS levels from 3 to 5), since most of the participants were identified at specialized centres for children with cerebral palsy (80% of the families). Similarly, our prevalence of pain and speech disability was slightly higher than that reported by other studies [51,52], probably due to the overrepresentation of children with greater impairments. These facts do not reflect the general distribution in the CP population, and the generalizability of the findings should be limited to children with the most severe impairments.

In conclusion, pain and, to a lesser extent, the ability to speak in children with CP can have an impact on the physical, social and psychological health of their families, although it does not seem to affect healthcare satisfaction. Periodic assessment and intervention of the family’s health and needs should be considered in the design of family-centred programs for children with CP.

## Figures and Tables

**Table 1:** Families’ sociodemographic characteristics.

Mother’s age (years; mean, SD)	41.54 (5.71)
Father’s age (years; mean, SD)	42.93 (6.46)
Number of siblings (*n*, %)	
One	7, 11.86%
Two	42, 71.19%
More than two	10, 16.95%
Marital status (*n*, %)	
Single	5, 8.48%
Married	45, 76.27%
Divorced	9, 15.25%
Education (*n*, %)	
Primary education	36, 61.02%
Secondary education	16, 27.12%
Higher education	7, 11.86%
Socioeconomic status (*n*, %)	
Low	11, 18.64%
Middle-low	27, 45.76%
Middle-high	20, 33.89%
High	1, 1.70%
Employment (*n*, %)	
Both parents full time employed	37, 62.71%
One parent half-time employed	13, 22.03%
Unemployed	9, 15.25%
Residence (*n*, %)	
Urban	14, 23.73%
Country	45, 76.27%

**Table 2:** Clinical characteristics of children with cerebral palsy. GMFCS = Gross motor function classification system (1 = walks without limitations, 5 = transported in a manual wheelchair) [35].

	*n*, %
Type of cerebral palsy	
Bilateral spastic	40, 67.80%
Unilateral spastic	4, 6.78%
Diskinetic	11, 18.64%
Ataxic	4, 6.78%
Cognitive impairment	
None	19, 32.20%
Mild	7, 11.86%
Moderate	3, 5.09%
Severe	30, 50.85%
Motor impairment (GMFCS)	
Level 1	7, 11.86%
Level 2	7, 11.86%
Level 3	12, 20.34%
Level 4	6, 10.17%
Level 5	27, 45.76%
Type of education	
Ordinary centre	47, 79.66%
Special centre	12, 20.34%

**Table 3:** Mean (standard deviation) and range of the different domains of the PedsQL^TM^ 2.0 Family Impact Module and the PedsQL^TM^ Healthcare Satisfaction Generic Module in every group of children.

	No Pain, Speech (N = 18)	No Pain, No Speech (N = 11)	Pain, Speech (N = 17)	Pain, No Speech (N = 13)
Family Impact Module				
Global scores				
Total impact	69.71 (20.91), 26.39–94.44	61.46 (12.52), 38.89–72.22	70.49 (13.44), 46.53–88.89	74.90 (14.21), 50.69–96.53
Parent health-related quality of life summary	79.11 (20.83), 37.50–100	62.71 (14.80), 42.50–75.00	73.91 (15.68), 48.75–96.25	78.57 (15.56), 50.00–100
Family functioning summary	75.00 (27.56), 0–100	72.45 (14.83), 53.57–92.86	73.81 (14.83), 46.43–92.86	83.67 (15.82), 57.14–100
Dimensions				
Physical functioning	79.72 (22.82), 25.00–100	58.33 (20.27), 33.33–83.33	64.38 (16.62), 37.50–95.83	76.79 (29.74), 16.67–100
Emotional functioning	76.67 (25.96), 10.00–100	56.43 (22.12), 20.00–80.00	61.50 (30.65), 0–100	63.57 (23.58), 30.00–100
Social functioning	80.42 (16.00), 43.75–100	65.48 (16.31), 37.50–87.50	71.25 (20.24), 43.75–100	83.93 (27.68), 37.50–100
Cognitive functioning	79.64 (25.53), 15.00–100	77.86 (20.59), 50.00–100	83.33 (11.99), 70–100	91.43 (20.56), 45.00–100
Communication	83.93 (22.38), 33.33–100	70.24 (30.89), 41.67–100	82.41 (17.40), 50.00–100	85.71 (11.50), 66.67–100
Worry	41.43 (35.91), 0–100	50.00 (26.93), 0–75.00	58.33 (13.23), 40–80	56.43 (22.86), 30.00–95.00
Daily activities	61.61 (34.13), 0–100	37.50 (27.00), 0–87.50	55.56 (25.09), 0–75.00	60.71 (34.93), 0–100
Family relationships	80.36 (29.32), 0–100	86.43 (16.26), 65.00–100	81.11 (15.77), 50.00–100	92.86 (9.51), 80.00–100
Healthcare Satisfaction Generic Module				
Global score	94.13 (25.50), 2.22–94.13	58.97 (15.14), 40.07–82.00	59.27 (24.63), 16.50–59.27	76.89 (15.49), 50.00–100
Information	54.60 (32.99), 0–95.00	55.00 (27.02), 25.00–90.00	60.83 (18.29), 40.00–100	70.29 (31.68), 25.00–100
Inclusion of family	66.00 (31.60), 0–100	45.83 (28.96), 0–75.00	54.44 (38.75), 0–100	85.71 (20.32), 43.75–100
Communication	63.27 (29.60), 0–100	49.00 (11.24), 35.00–60.00	63.04 (31.27), 5.00–100	63.97 (22.47), 40.00–100
Technical skills	57.51 (28.74), 8.33–100	75.00 (13.94), 66.67–100	54.38 (38.75), 0–100	88.33 (17.45), 60.00–100
Emotional needs	50.83 (29.47), 0–93.75	41.46 (31.05), 0–87.50	51.56 (40.35), 0–100	63.75 (21.07), 40.00–100
Overall satisfaction	79.22 (33.09), 0–100	87.50 (26.46), 58.33–100	83.33 (27.22), 25.00–100	89.29 (10.67), 60.49–100

**Table 4:** Statistical values of group comparisons in all the different dimensions of the Family Impact and Healthcare Satisfaction modules. Two-way ANOVAs, with PAIN (children with pain vs. children without pain) and SPEECH (children with speech ability vs. children without speech ability) as between-subject factors. * *p* < 0.05, ** *p* < 0.01.

	Main Effect PAIN	Main Effect SPEECH	Interaction PAIN × SPEECH
Family Impact Module			
Global scores			
Total impact	F = 1.39, *p* = 0.247	F = 0.10, *p* = 0.752	F = 1.107, *p* = 0.301
Parent health-related quality of life summary	F = 0.70, *p* = 0.408	F = 0.85, *p* = 0.363	F = 2.75, *p* = 0.108
Family functioning summary	F = 0.49, *p* = 0.488	F = 0.26, *p* = 0.613	F = 0.75, *p* = 0.392
Dimensions			
Physical functioning	F = 0.04, *p* = 0.838	F = 0.36, *p* = 0.555	F = 5.04, *p* = 0.031 *
Emotional functioning	F = 0.21, *p* = 0.653	F = 1.06, *p* = 0.311	F = 1.59, *p* = 0.215
Social functioning	F = 0.50, *p* = 0.486	F = 0.29, *p* = 0.865	F = 4.38, *p* = 0.044 *
Cognitive functioning	F = 1.43, *p* = 0.241	F = 0.19, *p* = 0.665	F = 0.47, *p* = 0.499
Communication	F = 1.12, *p* = 0.298	F = 0.62, *p* = 0.437	F = 1.66, *p* = 0.207
Worry	F = 0.24, *p* = 0.629	F = 0.89, *p* = 0.768	F = 0.89, *p* = 0.768
Daily activities	F = 0.65, *p* = 0.425	F = 0.79, *p* = 0.379	F = 1.89, *p* = 0.178
Family relationships	F = 0.24, *p* = 0.629	F = 1.46, *p* = 0.235	F = 0.15, *p* = 0.703
Healthcare Satisfaction Generic Module			
Global score	F = 0.96, *p* = 0.335	F = 0.88, *p* = 0.354	F = 1.73, *p* = 0.197
Information	F = 1.14, *p* = 0.294	F = 0.24, *p* = 0.629	F = 0.20, *p* = 0.657
Inclusion of family	F = 2.50, *p* = 0.123	F = 0.38, *p* = 0.540	F = 8.24, *p* = 0.007 **
Communication	F = 0.62, *p* = 0.437	F = 0.51, *p* = 0.482	F = 0.66, *p* = 0.423
Technical skills	F = 0.27, *p* = 0.609	F = 6.80, *p* = 0.014 *	F = 0.70, *p* = 0.410
Emotional needs	F = 1.06, *p* = 0.312	F = 0.16, *p* = 0.901	F = 0.93, *p* = 0.343
Overall satisfaction	F = 0.89, *p* = 0.768	F = 0.52, *p* = 0.478	F = 0.14, *p* = 0.907

## Data Availability

The data presented in this study are available on request from the corresponding author.

## Supplementary Materials

The following are available online at https://www.mdpi.com/2227-9067/8/2/87/s1, Table S1: Interview questions.
