# Supplementary material for: Pain and Communication in Children with Cerebral Palsy: Influence on Parents’ Perception of Family Impact and Healthcare Satisfaction
Source: Children (Basel). 2021 Jan 27;8(2):87. doi: 10.3390/children8020087 (PMC7912482; doi:10.3390/children8020087)

children

Supplementary Table S1. Interview questions.

FAMILY SOCIODEMOGRAPHIC DATA

Mother's age: .... years

Father's age: .... years

Marital status: ☐ single ☐ married ☐ divorced ☐ widow/er

Number of children: ....

Educational level: ☐ primary education ☐ secondary education ☐ higher education

Socioeconomic status: ☐ low ☐ middle-low ☐ middle-high ☐ high

Mother's employment: ☐ full time ☐ half time ☐ unemployed

Father's employment: ☐ full time ☐ half time ☐ unemployed

Residence: ☐ urban ☐ country

CHILD DATA

Sex: ☐ girl ☐ boy

Age: .... years

Type of education: ☐ ordinary center ☐ special center

Has the child speech ability? ☐ Yes ☐ No

Has your child chronic pain (pain lasting more than 3 months)? ☐ Yes ☐ No

Please rate how much pain you think your child is having **at the present time**:

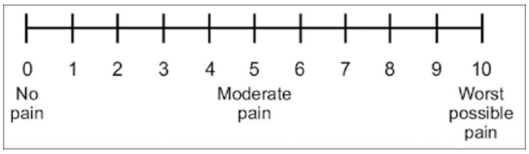

Please rate how severe **the worst pain** you think your chil had in **the past week** (7 days)

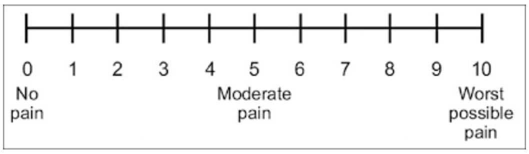

Please mark an X on the exact place where you think your child is having pain now. If there is more than one painful place, mark them '1', '2', '3', etc., starting with the most painful place as '1'.

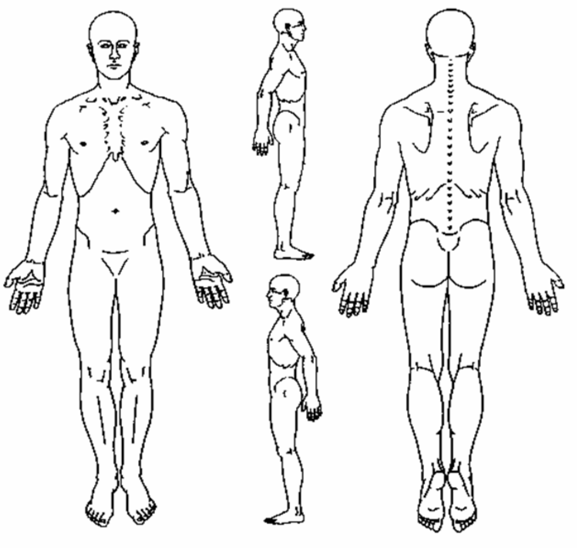

Supplement: Supplementary file 1 [file children-08-00087-s001.pdf]
